# High speed two-photon laser scanning stereomicroscopy for three-dimension tracking multiple particles simultaneously in three-dimension

**DOI:** 10.3389/fphot.2022.985474

**Published:** 2022-09-06

**Authors:** Xun Chen, Yang Li, Peng Chen, Hai Yao, Tong Ye

**Affiliations:** 1 Department of Bioengineering, CU-MUSC Bioengineering Program, Clemson University, Charleston, SC, United States; 2 School of Engineering Medicine, Beihang University, Beijing, China; 3 Department of Regenerative Medicine and Cell Biology, Medical University of South Carolina, Charleston, SC, United States

**Keywords:** two photon stereomicroscopy, high-speed volumetric imaging, 3D particle tracking, multiple particle tracking, multiplexing and demultiplexing

## Abstract

In this paper, we will describe a video rate two-photon laser scanning stereomicroscopy for imaging-based three-dimensional particle tracking. Using a resonant galvanometer, we have now achieved 30 volumes per second (frame size 512 × 512) in volumetric imaging. Owing to the pulse multiplexing and demultiplexing techniques, the system does not suffer the speed loss for taking two parallax views of a volume. The switching time between left and right views is reduced to several nanoseconds. The extremely fast view switching and high volumetric imaging speed allow us to track fast transport processes of nanoparticles in deep light-scattering media. For instance, in 1%-intralipid solution and fibrillar scaffolds, the tracking penetration depth can be around 400 μm.

## Introduction

Particle tracking has become a powerful tool to investigate molecular transport and biochemical dynamics in cells and tissues. To be able to track particles in tissues, commonly considered as scattering media, imaging methods with a high spatiotemporal resolution and a deep penetration depth are desired. For high spatiotemporal resolution imaging, camera (CCD or CMOS) based optical wide field microscopes would be a preferred choice because of the high imaging speed. However, the imaging depth of camera-based microscopies is limited by the mean free path (MFP, 1/scattering coefficient), which is a tissue dependent depth where scattering does not overwhelm imaging signals (Ntziachristos, 2010; Leigh et al., 2014). Confocal and multiphoton laser scanning microscopies are commonly used to overcome the MFP limit (Vasilis, Nature methods, 2010) (Ntziachristos, 2010). Note that two-photon scanned light-sheet (LSM) (Spille et al., 2012) or multifocal plane microscopy (MPM) (Ram et al., 2008) or selective plane illumination microscopy (SPIM) have been developed for particle tracking. However, these methods still use cameras as recording devices; the imaging depth is still at the level of the MFP.

Particle tracking in tissues also requires reporting particle displacements in three-dimensional coordinates. Most of current three-dimensional (3D) particle tracking techniques suffer from problems including 1) limited penetration depth [for instance, one-photon laser scanning microscope (Wells et al., 2010) and camera based optical wide field microscope (Ding and Li, 2016)], 2)poor temporal resolution [conventional galvo scanning method (Yang et al., 2016)], 3)inability to track multiple particles in one experiment [active feedback tracking methods (Spille et al., 2012; Perillo et al., 2015)], and 4)limited axial tracking range [engineered 3D point spread function tracking method (Huang et al., 2008; Shuang et al., 2016)].

To solve problems, two-photon (2p) laser scanning microscopy has become a standard method for deep lightscattering tissue imaging, but a few microscope developments for 3D particle tracking based on two-photon excitation have been reported. By using 2p laser scanning microscopy, the depth can be larger than mean free path. For example, optical phaselocked ultrasound lens (OPLUL) or tunable acoustic gradient (TAG) lens has achieved a fast continuous volumetric imaging (Kong et al., 2015; Hou et al., 2020) speed at tens of hertz in twophoton laser scanning microscopy. However, the continuous imaging method decreases exposure time, leading to lower S/N when it is applied to particle tracking.

More recently, engineered 3D point spread function (PSF) laser scanning microscopy has tracked fluorescent particles with 3D super resolution (Wang et al., 2017; Wang et al., 2020). However, this phase mask based engineered PSF method has a limited axial tracking range of 3 μm (Shuang et al., 2016). Moreover, two photon laser scanning microscopies using an active feedback tracking method (Ram et al., 2008; Ding and Li, 2016) provides high temporal resolution and high particle tracking localization precision, but cannot track multiple particles in one experiment. Our future application aims to investigate 3D dynamic transport of nanoparticles in biological tissues. Based on the ergodic hypothesis (Bel and Barkai, 2005) in particle tracking, we need to track multiple trajectories in one experiment.

We have previously reported on a two-photon laser scanning stereomicroscope at the speed of 1.4 volumes per second (Yang et al., 2016). Extended depth of field (EDF) can be used in twophoton laser scanning fluorescence microscopy to improve the volumetric imaging speed for observing fast particle tracking in three dimensions. By scanning extended beams with photomultiplier detection, we project particle motions in two views of a volume and recover the depth information. However, using conventional galvo scanning, the stereoscopic imaging approach with successive frames for left and right views resulted in a time delay of milliseconds between different views and tens of milliseconds between different volumes. For instance, Abbas at al. Developed Kilohertz frame-rate twophoton tomography using scanned line angular projection beam (Ding and Li, 2016). This destroys the simultaneity of stereoscopic imaging and decreases temporal resolution for particle tracking. Currently there is no single solution to all the above issues.

**FIGURE 1:**
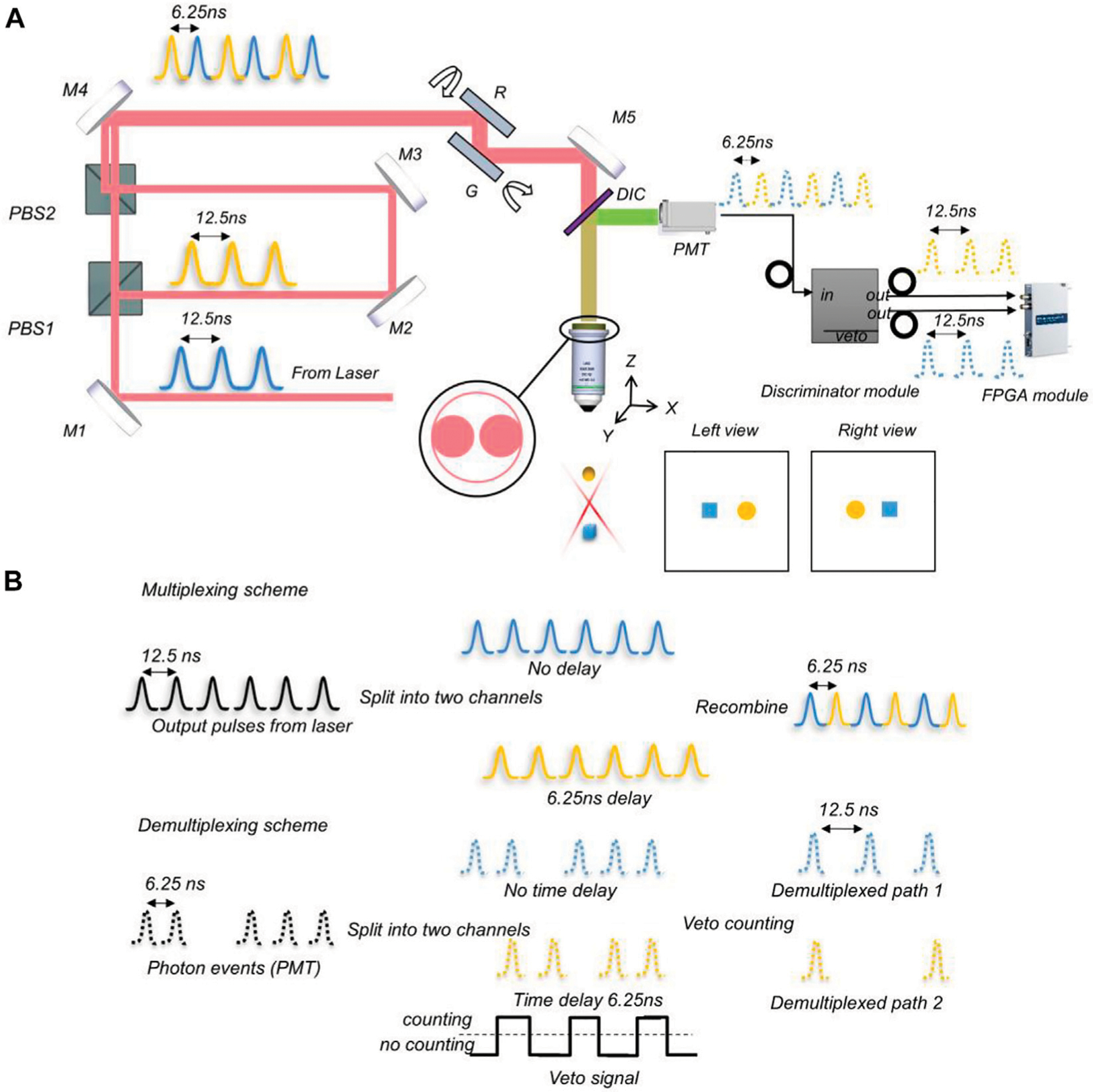
Schematic of vLSSM. (A) Setup of video rate two-photon laser scanning stereomicroscopy. M1-M5: Mirrors; DIC, Dichroic mirror; PBS, Polarizing beamsplitter; G, Galvo scanner; R, Resonant scanner; PMT, Photomultiplier tubes. Reflectance mirrors M2 and M3 keep two parallax beams in parallel. The *X*-axis resonant mirror and *Y*-axis Galvo mirror scan a projection volume by X-Y scanning. Two raw individual frames contain left and right views corresponding to two parallax beams respectively. (B)Multiplexing and demultiplexing scheme. In multiplexing, laser pulses split into two pathways, and pathway 2 is delayed. The 6.25ns delay in pathway 2 results in perfectly interweaved pulses upon recombination. In demultiplexing, PMT pulses are equally divided into two pathways. The second pathway is delayed by 6.25ns. A veto signal width of 6.25ns is applied to the two detection pathways resulting in two demultiplexed pulses streams.

To solve these problems, we report on video rate two-photon laser scanning stereomicroscopy (vLSSM, Figure 1A), a new approach that can achieve fast volumetric imaging based on a previous two-photon stereo-microscope. Due to multiplexing and demultiplexing technique, our system switches left and right views within several nanoseconds. The temporal identified views enable this system to deal with moving objects overlay. Herein, we demonstrated that vLSSM can track tens of particle’s motions at 30 Hz by using resonant scanning. The observation depth in intralipid solution and tissue-engineered fibrillar scaffolds can be around 400 μm with 2p laser scanning excitation. Tank et al. applied V shape extended PSF to volumetric two-photon imaging of neurons using stereoscopy (vTwINS) (Song et al., 2017) at 30 Hz. However, defining moving objects overlay in particle tracking was challenging with these methods. Our reported system can avoid this pitfall with two temporal separated parallax views.

## Methods

### Stereomicroscopy setup

The three essential requirements for constructing a stereomicroscope are the extended depth of field (EDF) to make a large volume of the sample in focus, two viewing angles to form a parallax, and an appropriate simultaneity of imaging of the two views to co-register the features and enable a successful depth perception. To follow the requirements, it is beneficial to generate a parallax by tilting Gaussian beams with a resonant mirror because of its fast-scanning speed. The *X*-axis resonant mirror (SC-30, Electro-optical products Corp) and *Y*-axis galvo mirror (GVS001 single-axis motor/mirror, GHS001 post adapter, Thorlabs), shown in Figure 1A, rotate synchronously and are kept in parallel during scanning. Since the scanning speed of the resonant mirror is about 8 kHz for small angles, the imaging speed can reach video rate (30 volumes/s). The Stereo-scanner (resonant and galvo scanner pairs) can replace the conventional X-Y galvo scanner in the two-photon microscope and delivers fast, stable, and flexible scanning behavior that the vLSSM needs. Another remarkable feature of vLSSM is the time delay between left and right views. This delay can be reduced to the nanosecond level by switching views pulse-by-pulse instead of line-by-line. Figure 1B shows the multiplexing and demultiplexing scheme which recombines extended focus beams between the left and right views and separates them into PMT pulses by a discriminator (ORTEC 935, ORTECT). As shown in Figure 1B, by using veto synchronization signals of 80 MHz from pulse laser, the discriminator differentiates the multiplexing fluorescent signals into two channels. The gating frequency of discriminator can be as fast as 80 MHz to handle two channels.

In order to achieve high-speed volumetric imaging, an ultrafast Ti:Sapphire laser (Chameleon Ultra, Coherent) was tuned to 864 nm for excitation. The galvo control waveform was generated by an analog output channel of a DAQ board (PCI-6363, National Instruments). The emitted fluorescence was collected by a high NA objective lens (N16XLWD, 0.8NA, Nikon). Then the fluorescence passed through the dichroic mirror (FF662-FDi01, Semrock, Rochester, NY) and a lowpass filter (FF01–665/SP-25, Semrock, Rochester, NY), and was finally focused onto PMT (PMC-100–4, Becker & Hickl GmbH). These PMT events are digitized with a high-speed digitizer (NI 5732, National Instruments). FPGA detection module (PXIe 7961R, National Instruments) assembled digital signals into images, and the synchronized resonant line waveform was detected by the digital input channel. The data acquisition from FPGA module simultaneously synchronizes with laser pulse. The PXI is a perfect platform to utilize the mechanical control and high-speed data acquisition that realtime imaging requires in our system. High-speed data acquisition and digital analog modules will be inserted in the PXI chassis. Based on Sciscan (Scientifica), the control program was written in LABVIEW (National Instruments).

### 3D Depth reconstruction and particle trajectory detection

**FIGURE 2:**
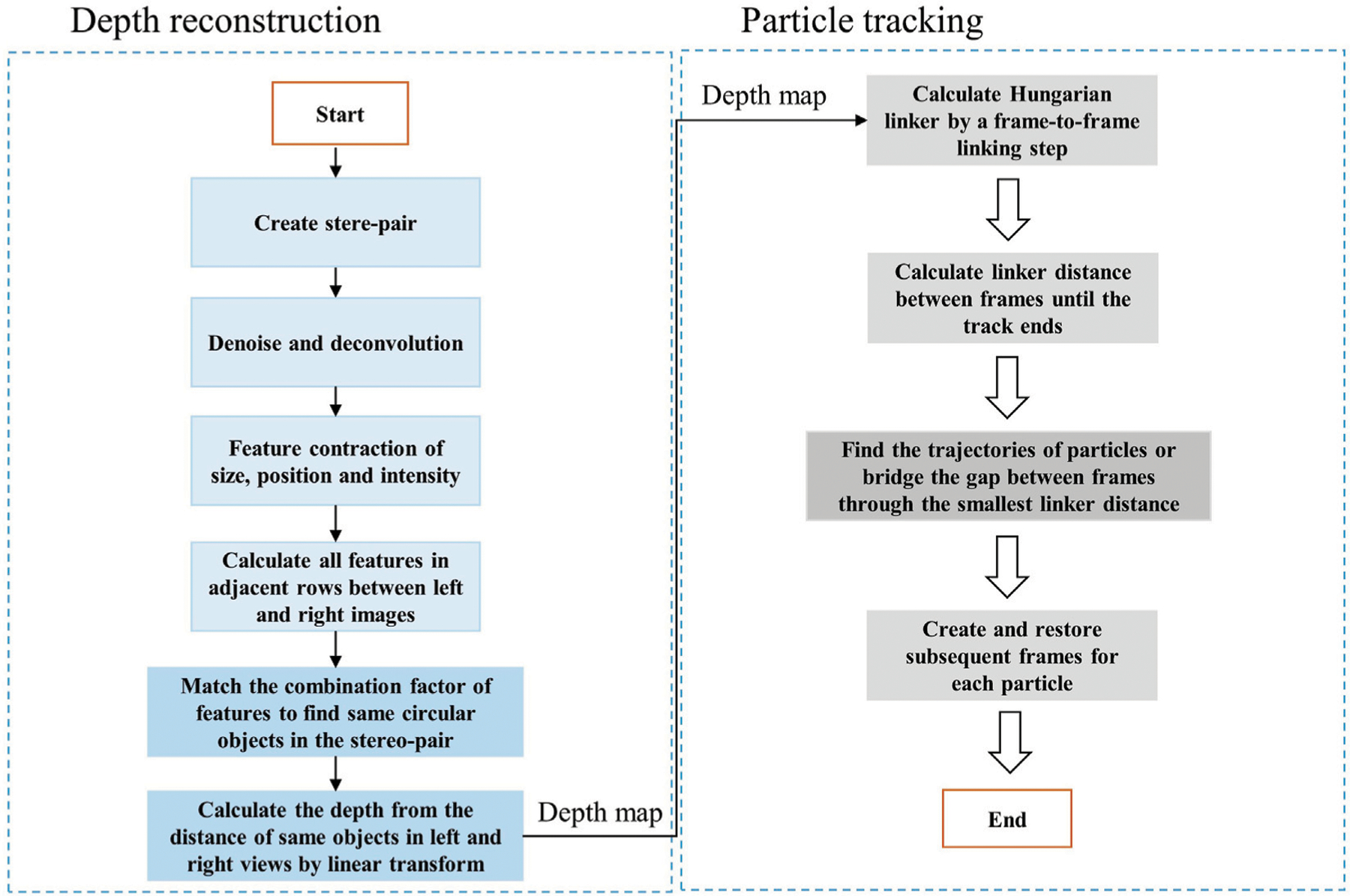
Flowchart of 3D depth reconstruction and particle trajectory algorithm.

To track the trajectory of particle’s motions in 3D, we need to first recover the stereoscopic depth of particles from stereo-images. In conventional two-photon laser scanning microscopy (TPM), the depth is directly related to different layers of a z-stack image. However, in vLSSM, the depth information is encoded in the stereo-pair. If the fluorophore distribution is sparsely dispersed in 3D and presents as recognizable features in EDF images, the depth information can be reconstructed by feature-based correspondence algorithms. As shown in Figure 2, the schematic in brief is that: 1) We first create the stereo-pair with left view and right view images. 2) We denoise the images with low-pass filter and deconvolved the images with Lucy-Richardson algorithm (Solomon and Breckon, 2011) to increase the contrast of particles. 3) The particles are then segmented by the adaptive threshold value. The morphology features such as simple geometric shapes, intensity, and position of single particles are extracted from left view and right view images. 4) We calculate all features in adjacent rows between left and right images, and find the best match based on combination factor of feature position, size and encircled energy. The circular Hough Transform (Davies, 2005) is used to find circular objects in the stereo-pair captured by vLSSM. 5) We calculate the depth of each matched particle from the distance of same objects in left and right views by linear transform with parallel projection geometry. The detail was described in our previous paper (Yang et al., 2016).

Tracking or particle linking is necessary in re-building the trajectories of one or several particles as they move along time. Their position is reported at each frame, but their identity is yet unknown; we do not know what particle in one frame corresponds to a particle in the previous frame. Tracking algorithms aim at providing a solution to this problem. As shown in Figure 2, the schematic in brief is that: 1) Hungarian linker (Liu et al., 2021; Stevens and Sciacchitano, 2021; Oleksiienko et al., 2022) starts by a frame-to-frame linking step, where links are first created between each frame pair. 2) Then, a second iteration is done through the data, investigating the linker distance between frames until the track ends. 3)Finding the trajectories of particles and bridging the gap between frames could be realized with minimizing the linker distance. If a track beginning is found close to a track end in a subsequent frame, a link spanning multiple frames can be created and restored. Source to target assignment is based on the famous Hungarian algorithm written in MATLAB (MathWorks) (Tinevez, 2022). The depth reconstruction and particle tracking algorithm run on the computer (Intel i9-7920x CPU). The overall 3D reconstruction and tracking process costs 5–10 min for each image with 30–50 particles.

## Results

### Localization precision of 3D reconstruction

For precise characterization of vLSSM, the whole excitation pathway was measured to have a focal volume of about 110 μm× 110 μm × 100 μm by scanning the tilted Gaussian beams at any position. The focal volume was measured by *in-situ* camera after objective. By moving the reflect mirror in *z*-axis by the digital stage, the axial FWHMs of the Gaussian foci is reconstructed with 100 μm. The *x*-axis and *y*-axis focal area is also calibrated by the digital stage by moving the resolution target with 110 μm × 110 μm. It is possible to change the FWHM (Full width at half maximum) of a beam such that the lateral resolution and the depth of field will be both tunable. Measured two-photon excitation PSF2p of Gaussian beams use small, tilted angles (≈28.1°).

**FIGURE 3:**
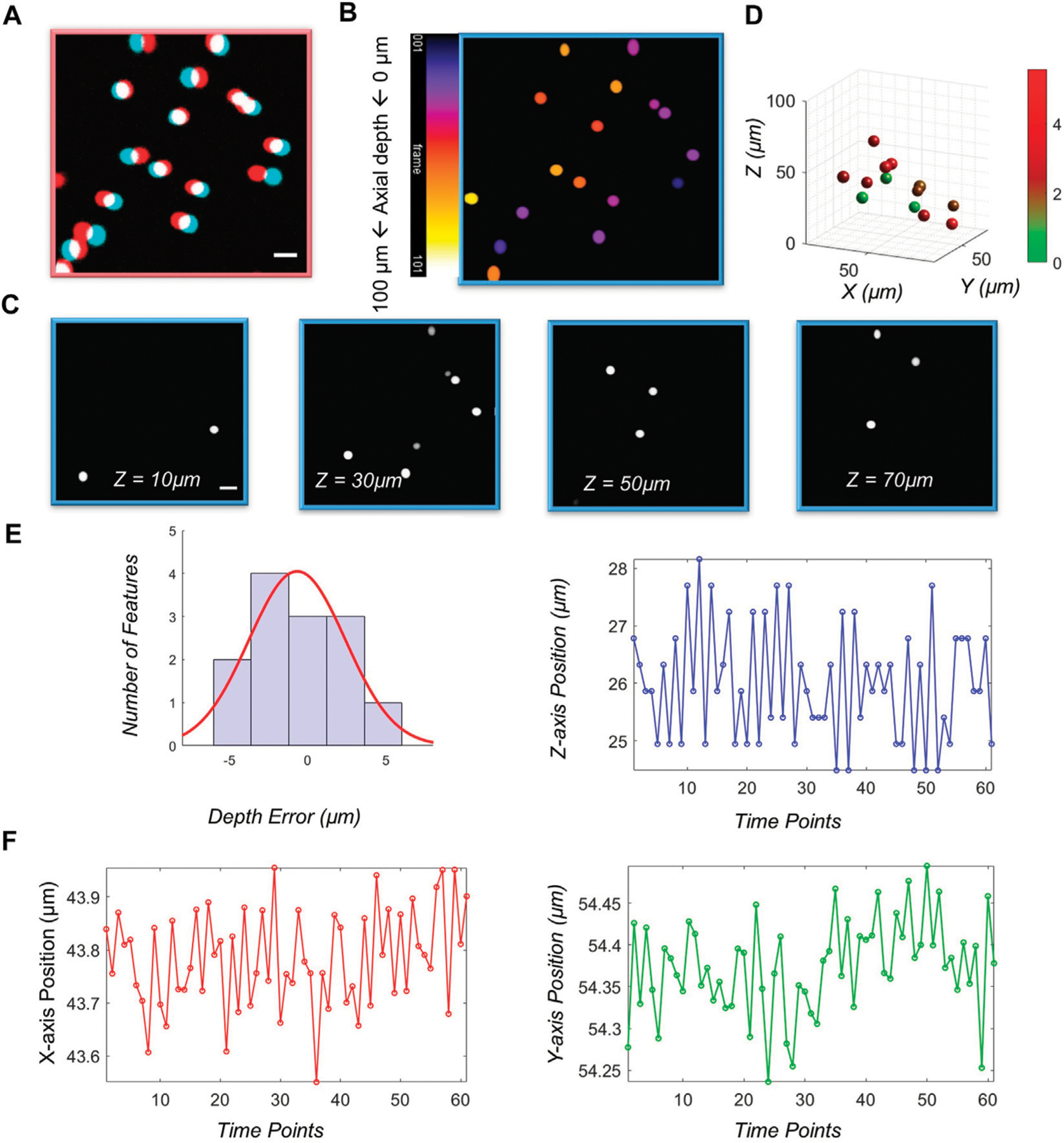
Characterizations of vLSSM. Stereo-pairs of (A) fluorescent beads (4.46 μm) were captured in stereo-mode. Red color means left view and cyan color means right view. (B) Sum projection of the stack along the *z*-axis. Color bar: 1 frame. (C) 4 selected images from an image stack consisting of 101 slices taken in standard two-photon mode. (D) 3D map of objects with their depth recovered from the stereo-pair in (A). Higher depth error indicated by red. (E) Histogram of the depth error with superposed normal distribution (red line). (F) Localization positions in *X*, *Y* and *Z* axes along a time sequence of 2 s.

We first demonstrated the imaging performance of the vLSSM using 4.46 μm diameter fluorescent beads (Cart21637, Polysciences) in Figure 3. The high-resolution stereo-pair frames clearly demonstrated VTPLSSM’s depth recovery ability due to two-photon excitation of Gaussian beams. With the featurebased depth reconstruction method, the fluorescent beads, shown with red and cyan channels in Figure 3A, can be simply recognized as circular objects with their sizes and locations determined by the circular Hough Transform. We identified 13 circular objects and obtained their disparities between left and right views. The relative depths of the objects were calculated according to their disparities with triangulation. To determine the accuracy of the recovered depths, we acquired the 3D stack of the same volume with conventional two-photon microscopy for establishing the ground truth values of the depth. The value can be used to evaluate the accuracy of the depths from the stereo-pair in Figure 3E. Figure 3D shows all 13 objects with their depth errors labelled with a color map that spans from green to red. Figure 3E gives the histogram of the depth error. About 42.9% of the objects are localized axially with depth error less than 2 μm, and 100% less than 5 μm. The depth error cannot be properly described by normal distribution, which is depicted as the superposed red line with its mean depth error 2.4836 ± 0.2 μm. To characterize localization precision of system, we measured the standard deviation of positions of particles (4.46 μm in diameter), shown in Figure 3F. It is 0.1 and 0.08 μm in the *X* and *Y* axis respectively and 1.1 μm in the *Z* axis along imaging sequences.

### Multiple particle tracking in 3D

**FIGURE 4:**
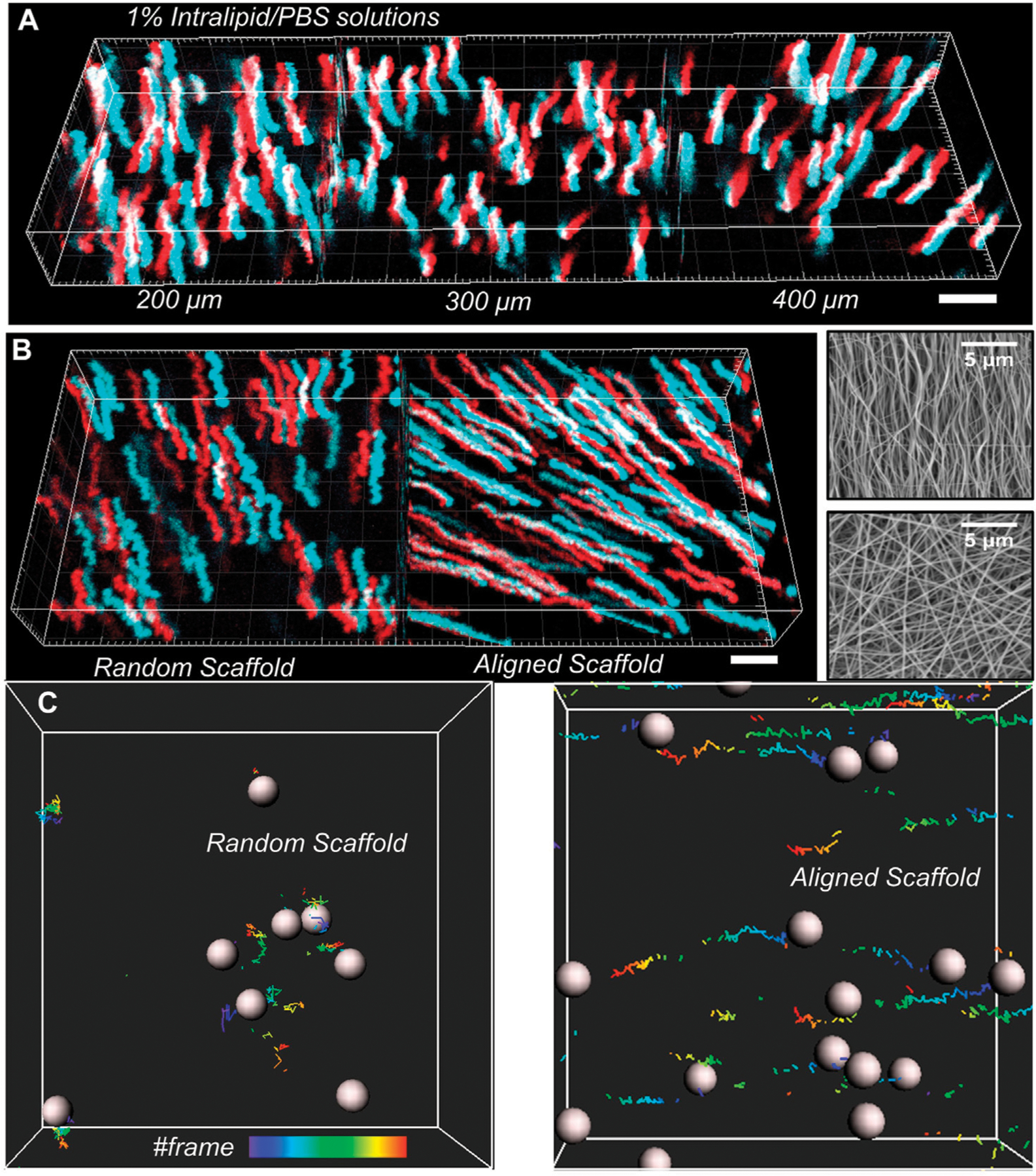
3D particle tracking reconstruction with (X, Y, T), red color means left view and cyan color means right view. (A) At the depth of 200, 300, 400 μm, the trajectories of nanoparticles in 1% intralipid/PBS solution with respect to time was tracked. (B) The total trajectories of nanoparticles in aligned and random gels were tracked with the projection of time flow. Scanning electron microscopy (SEM) images of fibrillar structure of both aligned and random gelatin with 3.0 k magnification. (C)Partial particle trajectories were reconstructed with frame series. Scale bar: 5 μm.

We then demonstrated the performance of the vLSSM for particle tracking in light-scattering media such as intralipid/phosphate-buffered saline (PBS) solution. The depth information is maintained without distortion due to high-speed volumetric imaging. A time sequence (4 s) of stereo-pairs was recorded and processed by a tracking algorithm. Figure 4A shows the trajectory of nanoparticles with a 500 nm diameter in 1% intralipid/PBS solution at three penetration depths: 200, 300, and 400 μm (see Supplementary Video S1). The corresponding square displacement with respect to time was calculated. There were 120 recorded time sequential steps, and corresponding imaging speed was 30 volumes/s with a frame size of 512 × 512 × 2 pixels. Driven by gravity, the particles fell with mean velocity (≈10 μm^2^/s) in the *Z*-axis. Compared to previous stereomicroscopy, the volumetric imaging speed of the vLSSM provides more time steps and allows the recovery of particle trajectories. The reduced scattering coefficient (μs’) and scattering coefficient (μs) of 1% intralipid/PBS solution under 864 nm excitation is around 0.85 and 3 mm^−1^ respectively (Grabtchak et al., 2012). By using 2p laser scanning microscopy, depth of imaging is expressed in terms of transport mean free path (TMFP = 1/μs’) and mean free path (MFP = 1/μs) (Leigh et al., 2014). The theoretical penetration depth range is from mean free path (0.3 mm) to transport mean free path (1.2 mm). Compared to camera based wide-field microscopy, the theoretical penetration depth range is limited less than mean free path (0.3 mm). The penetration depth of the vLSSM in 1% intralipid/PBS can be around 0.4 mm.

We then tracked nanoparticle (500 nmin diameter) transport in both randomly distributed and aligned gelation scaffolds (see Supplementary Video S2), which were fabricated by the electrospinning (ES) machine at depth of 400 μm shown in Figure 4B. The imaging processing and analysis are the same as in intralipid/PBS solution. The transport behavior of particles in tissue-engineered materials depends on the size of particles and fibrillar orientation. During the material preparation, the fibrillar orientation is controlled by the rotation speed of the electrical spinning motor.

From scanning electron microscopy (SEM) images shown in Figure 4B, we measured the fibrillar orientation distribution. The analysis algorithm is based on DiameterJ, an open-source plugin created for ImageJ. In aligned scaffolds, the peak orientation direction is 90° with respect to the *X*-axis at normalized frequency of 0.05. In fibrillar randomly distributed scaffolds, the peak orientation direction is 40° with respect to the *X*-axis at normalized frequency of 0.02. Because of structural sliding and gravity driving in aligned gelation scaffolds, the particles migrated along the fibrillar aligned direction. In random distributed scaffolds, the particles migrated randomly, in no specific direction.

## Discussion and conclusion

The current sensitivity and localization precision of our vLSSM cannot follow the nanoparticles (smaller than 500 nm) in deep scattering media. However, the system can be improved by following certain strategies with higher tracking localization precision: increasing the collection efficiency of system, increasing exposure time of particle objects, and increasing the brightness of tracked fluorescent particles. The microscopic collection efficiency is related to quadratic numerical aperture (NA). Through increasing the objective NA, the microscopic sensitivity can be highly improved. However, the major drawback of this approach is that the *z*-axis size of the focal spot also decreases. With a higher numerical aperture, the same system has a smaller DOF (Thériault et al., 2014; Zong et al., 2015). Moreover, through decreasing scanning scale, we can decrease scanning speed. The decrease of scanning speed leads to increased exposure time of particle objects. The brightness of particles depends on excited fluorescent core materials such as polymer, carbon, and inorganic sulfide with stabilization ligands. Generally, the brightness of quantum dots composed of inorganic sulfide with stabilization ligands are better than polymeric spheres, that is, we used in this paper. However, the quantum dot is the lack of both size-dependent emission wavelengths and toxicity compared to carbon dots (Cao et al., 2012; Liu et al., 2015). In future, we may track 100–500 nm carbon dots in live deep tissues. In summary, all above strategies has the potential to overcome 500 nm scale limitation using our tracking method in deep biological tissues.

In this paper, we have developed a video-rate two-photon laser scanning stereomicroscopy for 3D particle tracking using only two parallax frames. By stereoscopically scanning extended Gaussian beams in the excitation pathway, the particle depth could be reconstructed with 1.1 μm *z*-axis localization precision. We demonstrated the ability of vLSSM to track multiple nanoparticles in three dimensions in deep intralipid medium, aligned, and random distributed scaffolds. The fiber alignment was highly related to the tracked particle trajectories.

With resonant-galvo scanners, the system can achieve the speed of thirty volumes per second, and high S/Nto tackmultiple trajectories in three dimensions even in deep light-scattering media. Moreover, with the superior penetration ability of two-photon excitation, it is possible to capture the fast dynamic events in deep biological tissues by using two-photon stereomicroscopy. The stereoscopic technique described herein is anticipated to be implemented as an add-on imagingmode on a standard two-photon fluorescence microscope to meet various imaging applications. The three-dimensional fluorescent molecular motions in deep biological tissues will be directly viewed in real-time by wearing 3D glasses.

## Supplementary Material

Supplementary Video 1

Supplementary Video2

## Data Availability

The raw data supporting the conclusions of this article will be made available by the authors, without undue reservation.
